# Multidisciplinary Treatment Approach for Perforated Internal Root Resorption: Three-Year Follow-Up

**DOI:** 10.1155/2019/5848272

**Published:** 2019-09-19

**Authors:** Sinem Yıldırım, Mesut Elbay

**Affiliations:** ^1^Department of Pediatric Dentistry, Faculty of Dentistry, Istanbul Okan University, Istanbul, Turkey; ^2^Department of Pediatric Dentistry, Faculty of Dentistry, Kocaeli University, Kocaeli, Turkey

## Abstract

Internal root resorption (IRR) is described as a resorptive defect of the internal aspect of the root caused by odontoclastic activity associated chiefly with chronic pulp inflammation and trauma. An important variation to consider is the presence of a root perforation, although it is rare. This paper defines the use of CBCT in the diagnosis and combined nonsurgical and surgical multidisciplinary management and follow-up of a maxillary central incisor with perforating IRR in a 9-year-old female patient. At 3-year follow-up, clinical and radiographic findings of the case were satisfactory.

## 1. Introduction

Internal root resorption (IRR) is defined as the progressive devastation of intraradicular dentin and dentinal tubules throughout the middle and apical thirds of the canal walls as a consequence of odontoclastic activity. Odontoclastic activity is associated mainly with chronic pulp inflammation and trauma [1]. Various etiological factors have been considered for the loss of predentin, with trauma being the most advocated [2, 3].

IRR is normally asymptomatic and often recognized clinically through routine radiographic investigations. However, when the resorption is predominantly expanding, the tooth may at least be partially vital and may indicate signs typical of pulpitis. Conventional radiographic images provide a two-dimensional display of a three-dimensional (3D) structure, which may lead to interpretation errors. For that reason, 3D evaluation of the resorptive zone with cone beam computerized tomography (CBCT) ensures crucial knowledge for early disease recognition and treatment methods. CBCT provides information such as the extent, form, and nature of the lesion including root perforations [4].

Critical to the clinical use of therapies aimed at controlling IRR is the correct diagnosis of the localisation and severity of the resorptive area [3]. In cases without perforation, eradication of the resorptive lesion from the root canal should be carried out to avoid further loss of healthy tissue. However, if the IRR has extended the external root surface, a track shows up between the root canal and the periodontal space is present and destruction of the contiguous periodontal tissues may occur. This situation makes the treatment more complicated and may negatively influence the long-term endodontic outcome of a tooth [5, 6].

One of the biocompatible and bioactive materials that can repair this perforation is mineral trioxide aggregate (MTA). In recent years, it has been shown in the literature that MTA is successful in the surgical and nonsurgical treatment of IRR [7, 8]. The present case report of a 3-year follow-up defines the use of CBCT in the recognition and combined nonsurgical and surgical treatment of perforating IRR in a maxillary central incisor.

## 2. Case Report

**Figure 1:**
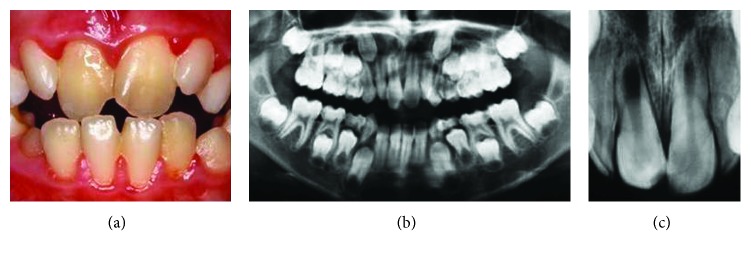
(a) Intraoral view of central incisors. (b) Panoramic radiograph. (c) Periapical radiograph before treatment.

A 9-year-old female patient applied to the Pediatric Dentistry Clinic owing mild pain correlated with her maxillary right central incisor. The medical background of the patient was noncontributory. She had no history of traumatic injury or orthodontic treatment. Clinical investigation revealed that the tooth was slightly tender to percussion and responded negatively to electrical/thermal vitality tests. Probing revealed no periodontal pocketing around the tooth, and mobility was within physiological limits. The tooth had no caries and restoration. She had transverse maxillary deficiency and the tooth 11 was in crossbite (Figure 1(a)). Radiographic examination revealed a well-circumscribed, quite oval radiolucent area in the middle third of the root (Figure 1(c)). No periapical radiolucency was detected. Depending on the clinical and radiographic evaluations, a diagnosis of IRR in tooth number 11 was made and endodontic treatment of tooth number 11 was suggested to the patient.

**Figure 2:**
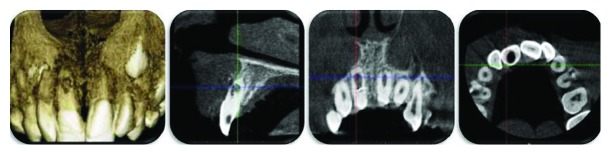
Pretreatment CBCT images.

The primary treatment plan involved endodontic treatment. Then, written consent was attained from the parents and patient; local anesthesia was administered accordingly. The access cavity was prepared on the palatal surface using high-speed round diamond burs (Kendo, VDW GmbH, Germany) under continuous water irrigation, and the pulp tissue was removed. After preparation of the access cavity, the coronal part of the pulp was found to be necrotic, while bleeding was induced on the middle part of the pulp. A communication between the middle third of the root surface and the buccal periodontium was observed. Buccal root surface was thought to be perforated. Further evaluation was conducted using a CBCT scan to investigate the position and borders of the resorption area (Planmeca ProMax 3D Max; Planmeca Oy, Helsinki, Finland). The CBCT scan was taken with a lead thyroid collar and a lead apron using a limited field of view at the smallest volume at 96 kV, 8 mA, and 200 *μ*m of voxel size. Three-dimensional images were reconstructed, and the images were analyzed using viewer software (Romexis; Planmeca Oy, Helsinki, Finland). Axial, sagittal, and coronal CBCT cross-sections approved the resorption area in the middle and apical third of the root canal which had perforated the buccal root surface (Figure 2). Also, the resorptive process had invaded the buccal cortical plate of the bone. Then, the treatment plan was revised. The probability of preventing the maxillary right central incisor was regarded through a combination of treatments: nonsurgical root canal therapy to extirpate the necrotic pulp and disinfect the root canal system, followed by surgical treatment to uncover the resorptive defect, and, finally, the resorptive defect was filled with white MTA.

**Figure 3:**
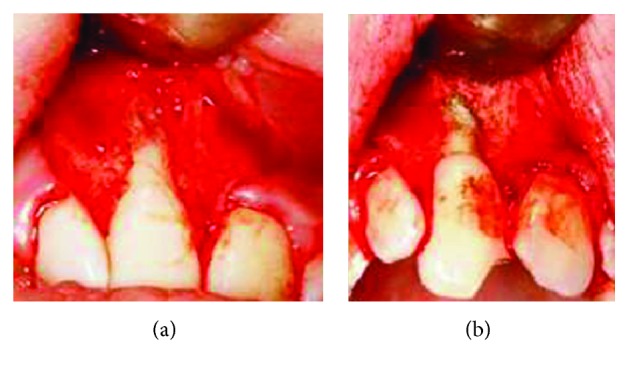
(a) Appearance of the perforation defect. (b) MTA sealing perforation defect.

For nonsurgical root canal treatment, the working length was determined using an electronic apex locator (Tri Auto ZX; Morita, Tokyo, Japan) and a periapical radiograph was taken. The chemomechanical preparation was applied using the step back technique with 1% sodium hypochlorite (NaOCl) (Wizard; Rehber Chemistry, Istanbul, Turkey) and 17% EDTA solution (Wizard; Rehber Chemistry, Istanbul, Turkey). The root canal was gently irrigated by irrigation needle having two lateral vents oriented to the opposite side of the perforation area in order to avoid the NaOCl extrusion. After paper-point drying of the root canals (MMPP; Diadent, Canada), calcium hydroxide paste (Metapaste; Meta Biomed, Korea) was placed into the canal. After that application, the access cavities were sealed temporarily with Cavit (3M ESPE AG; Seefeld, Germany). Until the tooth was asymptomatic, calcium hydroxide paste was changed once a month. Also, the left maxillary central incisor was necrotic and required root canal treatment. Nearly three months after initiating the treatment, endodontic therapy and coronal restoration of the left maxillary central incisor were completed. When tooth number 11 became asymptomatic, the canal was obturated using white MTA (Angelus; Londrina, PR, Brazil). At the same time, a flap that made visible the granulation tissue and the bone destruction was elevated (Figure 3(a)). The granulation tissue was eliminated, and the irregular verges of the perforation area were planed with a bur attached to a straight surgical handpiece. MTA material was prepared according to the manufacturer's instructions and placed with a MTA carrier (Figure 3(b)). MTA was securely condensed with the use of a plugger and wet cotton pellets. The patient's own bone fragments was fixed over the MTA to fill the cavity of the bony defect. The flap was sutured, and the patient was recalled 1 week later for suture removal.

**Figure 4:**
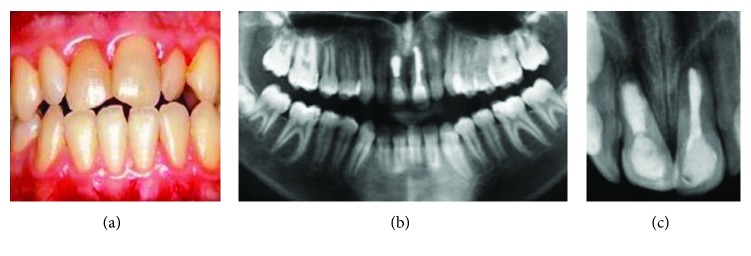
(a) Intraoral view of central incisors after 3 years. (b, c) Panoramic and periapical radiograph at 3-year follow-up.

The patient was scheduled for follow-up appointments for every 6 months. At 6 months and 3 years, the teeth were asymptomatic without gingival inflammation. Radiographs showed normal healing of the periapical areas (Figure 4).

## 3. Discussion

In this case report, clinical inspection revealed that both the right and left maxillary central incisors were exempt from caries or restorations. Also, the patient did not assert any traumatic injury. Patient had transverse maxillary deficiency and the teeth (11-21) were in crossbite. For this reason, it can be estimated that the traumatic occlusion could have commenced the resorptive period in this patient. Root resorption succeeding orthodontic teeth movement has been well demonstrated [8, 9]. But no literature on IRR due to traumatic occlusion has been found.

Traumatic occlusion may interfere with periodontal health owing to affect tooth mobility and the periodontal probing pocket depths. Additionally, it can bring about morphofunctional changes such as decline in collagen fibres, disorientation, and increased osteoclastic activity. Odontoclasts are the cells that resorp dental hard tissues and are mophologically similar to osteoclasts. Osteoclasts and odontoclasts resorb their target tissues in a similar manner. Both cells have possession of analogue enzymatic properties and both build resorption depressions specified Howship's lacunae on the surface of the mineralized tissues [10].

Patel et al. [11] conducted their research on patients with internal and external resorptions and concluded that although the periapical radiography is an acceptable diagnostic device, CBCT had more precision in the diagnosis of the internal resorption and, consequently, this method could enhance the chance and probability of a correct treatment. However, justification of the use of CBCT in children is particularly significant because of the higher risks associated with exposure in children [12, 13]. The smallest volume size in line with the condition should be picked by virtue of reduced radiation dose. In the present report, CBCT scan was not the first choice of imaging but confirmation of clinically suspected buccal perforation was necessary, so CBCT scan was taken using a limited field of view at the smallest volume with the radiation beam collimated to the restricted area. Considering that there was no interference with the scan, a lead thyroid collar was also used with a lead apron to reduce thyroid exposure.

One of the most important factors affecting the prognosis of IRR treatment is the presence of perforation. Close proximity of the perforation to the gingival sulcus can lead to the contamination of the perforation with bacteria from the oral cavity through gingival sulcus. If the perforation area is large and not treated immediately, the proximity to the epithelial attachment is critical, and apical migration of the epithelium to the perforation area will create a periodontal defect [14]. In this case, it was decided to seal the perforation area with MTA by surgical approaches in order to prevent the occurrence of additional complications mentioned above.

Vitality of the apical part of the resorption is another important factor for the prognosis of IRR treatment. Regenerative endodontic treatment (RET) is an alternative treatment approach in IRR cases which is based on the concept that multipotent stem cells from the apical area are able to induce pulp regeneration in immature permanent teeth. RET in mature teeth is challenging compared with that in immature teeth because of the lower amount of progenitor cells present and narrower apical pathways for cell migration [15]. In the present case, RET was not preferred considering mature apex.

Remineralization therapy with calcium hydroxide, which forms a hard tissue matrix against which to condense the root-filling material, has been advocated by Benenati [16] in the treatment of perforating IRR. However, calcium hydroxide has a number of limitations including variable treatment time ranging from 5 months to 20 months, an increased risk of tooth fracture, and poor patient compliance with follow-up due to the extended treatment time, all of which can affect treatment outcomes [17].

When surgical and nonsurgical treatments are not possible or have a long prognosis, intentional replantation (IR) is an option to save the tooth. IR should be considered a viable alternative to extraction. Recent case reports have demonstrated that with good case selection, IR can be a reliable and predictable procedure [18, 19]. Comparing success rates of IR is problematic as case selection is critical; moreover, the variability in tooth type and follow-up time creates confusion.

In the present case, during root canal shaping, the root canal was gently irrigated using 1% NaOCl. NaOCl is a widely used irrigation solution because of its enhanced antimicrobial activity and organic tissue dissolution capacity for the elimination of necrotic and granulation tissues from internal resorption cavities [20]. As a matter of precaution, a lower concentration of NaOCl solution was preferred as Kaval et al. [21] used in their case, to protect periradicular tissues from its toxic effects [22].

In the vast majority of the prior studies, the filling material has been applied after reparation of the perforation defect [6, 23, 24]. From another point of view, Yildirim and Dalci [25] sealed an iatrogenic root perforation with MTA after the root canal was filled with gutta-percha and AH plus sealer. In this case, we also preferred sealing the root canal completely with MTA before management of the buccal root resorption because filling the root canal after placement of MTA involves the risk of displacing MTA from the perforation site during condensation of filling materials.

Aslan et al. [26] studied stress distributions in the case of internal resorption when root canals were completely filled with MTA or MTA gutta-percha combinations. They reported that both MTA and MTA-gutta-percha combination were not different in terms of usage in clinical practice and could be used in both methods if the IRR cavity was obstructed. We also preferred to fill the root canal completely with MTA. Considering the thin and weakened tooth structure in the resorptive defect, a bioactive material was needed to reinforce the tooth and thereby enhance the prognosis of the tooth.

## 4. Conclusion

This case report demonstrates the benefits of utilizing CBCT in the assessment and management of perforating internal root resorption. A multidisciplinary approach is necessary for a good long-term prognosis of challenging cases such as this. Additionally, the clinician must have adequate knowledge of advanced diagnostic and treatment modalities for a successful management of perforated root canals due to IRR.
